# Downregulation of microRNA-182-5p contributes to renal cell carcinoma proliferation via activating the AKT/FOXO3a signaling pathway

**DOI:** 10.1186/1476-4598-13-109

**Published:** 2014-05-17

**Authors:** Xin Xu, Jian Wu, Shiqi Li, Zhenghui Hu, Xianglai Xu, Yi Zhu, Zhen Liang, Xiao Wang, Yiwei Lin, Yeqing Mao, Hong Chen, Jindan Luo, Ben Liu, Xiangyi Zheng, Liping Xie

**Affiliations:** 1 Department of Urology, First Affiliated Hospital, School of Medicine, Zhejiang University, Qingchun Road 79, Hangzhou 310003, Zhejiang Province, China

**Keywords:** Renal cell carcinoma, Proliferation, Microrna-182-5p, FLOT1

## Abstract

**Background:**

Emerging evidence has suggested that dysregulation of miR-182-5p may contribute to tumor development and progression in several types of human cancers. However, its role in renal cell carcinoma (RCC) is still unknown.

**Methods:**

Quantitative RT-PCR was used to quantify miR-182-5p expression in RCC clinical tissues. Bisulfite sequencing PCR was used for DNA methylation analysis. The CCK-8, colony formation, flow cytometry, and a xenograft model were performed. Immunohistochemistry was conducted using the peroxidase and DAB methods. A miR-182-5p target was determined by luciferase reporter assays, quantitative RT-PCR, and Western blotting.

**Results:**

miR-182-5p is frequently down-regulated in human RCC tissues. Epigenetic modulation may be involved in the regulation of miR-182-5p expression. Enforced expression of miR-182-5p in RCC cells significantly inhibited the proliferation and tumorigenicity *in vitro* and *in vivo*. Additionally, overexpression of miR-182-5p induced G1-phase arrest via inhibition of AKT/FOXO3a signaling. Moreover, FLOT1 was confirmed as a target of miR-182-5p. Silencing FLOT1 by small interfering RNAs phenocopied the effects of miR-182-5p overexpression, whereas restoration of FLOT1 in miR-182-5p -overexpressed RCC cells partly reversed the suppressive effects of miR-182-5p.

**Conclusions:**

These findings highlight an important role for miR-182-5p in the pathogenesis of RCC, and restoration of miR-182-5p could be considered as a potential therapeutic strategy for RCC therapy.

## Background

Renal cell carcinoma (RCC) is the most common type of adult kidney cancer, and clear cell RCC represents the most common renal cancer histology. The incidence and mortality rates of kidney cancer have increased in recent years, with an expected 271,000 newly-diagnosed cases and 116,000 deaths in 2008 worldwide [1]. About one-third of RCC patients are diagnosed with metastatic disease and up to 50% of patients develop metastatic disease [2] with a very poor prognosis because of the refractory nature of RCC to the current treatment regimens [3]. Therefore, it is crucial to identify novel therapeutic targets, including non-coding RNAs (ncRNA) in RCC, to develop more effective treatment options for this fatal disease.

MicroRNAs (miRNAs) are a class of small, endogenous, noncoding RNAs that are approximately 22 nucleotides in length and make up a novel class of gene regulators [4]. It has been firmly established that miRNAs regulate various cellular processes such as cellular differentiation, development, proliferation, apoptosis and metabolism [5,6]. Recent evidence indicates that the aberrant regulation of miRNAs plays an important role in RCC pathogenesis. Several studies profiling miRNA expression in RCC have identified a number of differentially expressed miRNAs though no consensus has been reached [7-11]. In addition, a series of miRNAs (including miR-34a, miR-145, miR-205, miR-708, miR-1285 and miR-1826) have been shown to modulate the viability, proliferation, invasion and metastasis of RCC cells [12-17].

MicroRNA-182-5p is a member of the miR-183 family which includes miR-96, miR-182 and miR-183. These miRNAs are coordinately expressed from a single genetic locus located at human chromosome 7q32.2. Most previous studies suggested an oncogenic role for miR-182-5p in various types of human cancers, including prostate cancer, breast cancer, bladder cancer, liver cancer, colon cancer, cervical cancer, ovarian cancer, and glioma [18-25]. However, three studies illustrated that the ectopic expression of miR-182-5p inhibits the cell growth of posterior uveal melanoma, lung cancer and stomach cancer by targeting MITF, BCL2, cyclin D2, RGS17 and CREB1 [26-28]. Therefore, defining the function of miR-182-5p is complicated because it can be an oncogene or a tumor suppressor in the context of different cancers. To the best of our knowledge, the biological function of miR-182-5p in RCC is not yet well understood. In this study we observed the frequent downregulation of miR-182-5p in human RCC tissues. In addition, for the first time, we found that miR-182-5p could suppress the proliferation and tumorigenicity of RCC cells by targeting FLOT1.

## Results

### miR-182-5p is down-regulated in RCC

**Figure 1:**
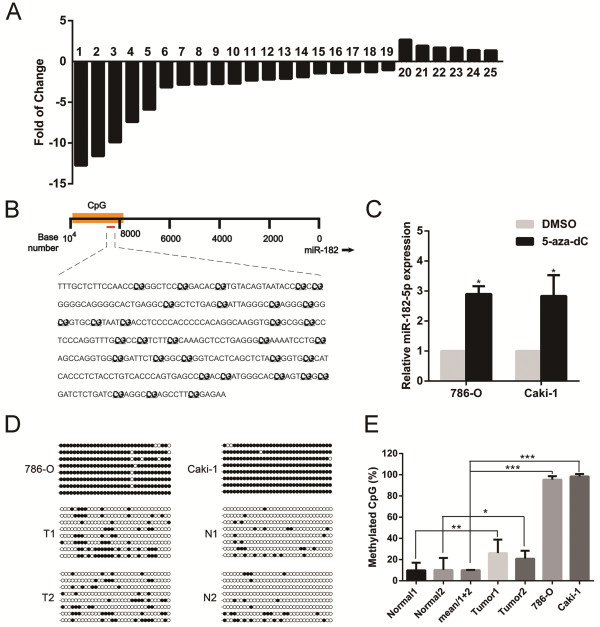
**miR-182-5p is down-regulated in RCC and regulated by DNA methylation. (A)** The relative expression levels of miR-182-5p in individual 25 pairs of RCC tissues were presented as the fold change of miR-182-5p referred to the corresponding normal tissues. **(B)** The regions analyzed by BSP are indicated. **(C)** DNA methylation inhibitor 5-aza-dC stimulated the expression of miR-182-5p compared with DMSO treated samples. Error bars represent the S.D. from three independent experiments. **(D)** and **(E)** The promoter of miR-182-5p was hypermethylated in RCC cell lines and tissues. The open and filled circles represent the unmethylated and methylated CpGs, respectively. Error bars represent the S.D. from eight randomly chosen colonies. ***P < 0.001; **P < 0.01; *P < 0.05.

As indicated by the previous published datasets, miR-182-5p was in a down-regulated expression pattern in RCC compared with normal renal tissue [29,30]. To further validate the expression pattern of miR-182-5p in RCC, we quantified the expression levels of miR-182-5p in 25 pairs of human RCC tissues and adjacent non-tumor tissues by qRT-PCR (Figure 1A). The relative expression of miR-182-5p was normalized to an endogenous control (U6 RNA). The results showed that the expression level of miR-182-5p was generally lower in tumor tissues compared to matched non-tumor tissues (19 out of 25 exhibited a down-regulated pattern). Thus, we speculated that miR-182-5p might be a putative tumor suppressor in RCC.

### Cancer-specific methylation pattern of CpG islands associated with miR-182-5p in RCC cells

Transcriptional regulation plays a crucial role in miRNA expression. We first predicted the presence of CpG islands in the upstream sequence of pri-miR-182. The miRStart database [31] (http://mirstart.mbc.nctu.edu.tw/home.php) predicted that the transcription start site (TSS) of miR-182-5p may be located 9729 upstream of the precursor. Then, the CpG Island Searcher program (http://www.urogene.org/methprimer/) helped us to identify a prominent CpG island located 8–10 kb upstream of miR-182 (Figure 1B). Liu et al. previously reported that this CpG island was exclusively methylated in melanoma cells and may be involved in the regulation of miR-182 expression [32]. To clarify the possible roles of the epigenetic mechanism of miR-182-5p silencing in RCC cell lines, we treated 786-O and Caki-1 with 5-Aza, a methyltransferase inhibitor. We found that the treatment of cells with 5-Aza significantly elevated the expression of miR-182-5p in both cell lines (Figure 1C), indicating the existence of epigenetic regulation.

We next conducted sodium bisulfite sequencing assay to evaluate the methylation status of the predicted CpG island. The results illustrated that the methylation levels were significantly higher in RCC cell lines (786-O and Caki-1) and tissues, compared with para-cancer normal kidney tissues (Figure 1D and E). While 95.5% and 98.5% of CpGs were methylated in 786-O and Caki-1 cells, respectively, only 9.9% and 10.2% of CpGs were methylated in normal samples 1 and 2. The methylation levels of tumor samples 1 and 2 (26.1% and 20.8%, respectively) were relatively low compared with 786-O and Caki-1 cells. Moreover, the treatment of cells with 5-Aza apparently reduced the methylation level of this CpG island (Additional file 1: Figure S1), which further indicated that this epigenetic modulation could be involved in the regulation of miR-182-5p expression.

### Overexpression of miR-182-5p inhibits the proliferation and tumorigenicity of RCC cells *in vitro and in vivo*

**Figure 2:**
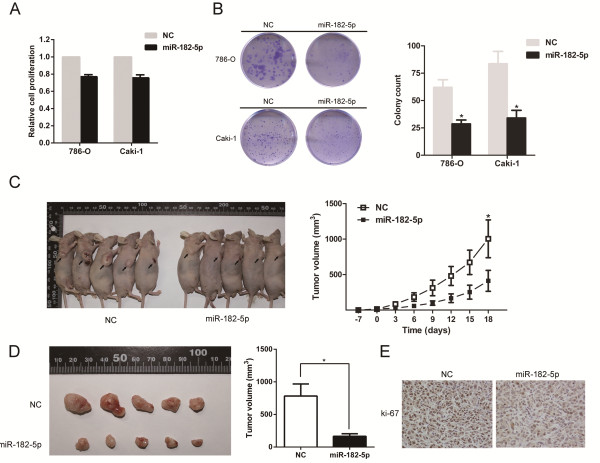
**Effect of miR-182-5p in regulating RCC cells proliferation. (A)** CCK-8 assay. The relative cell viability of the miR-182-5 transfected group was significantly lower than that of NC transfected. **(B)** Colony formation assay (Representative wells were presented). The colony formation rate was significantly lower for miR-182-5p treated group compared with NC treated group. Error bars represent the S.D. from three independent experiments. **(C)**, **(D)** and **(E)** Tumor xenograft model. The tumor volumes and the growth curves indicated that tumor in miR-182-5p group was in a significant slower growth pattern. Decreased Ki-67 expression was also detected in miR-182-5p treated tumors. Error bars represent the S.D. from five nude mice. *P < 0.05.

We transfected the RCC cell lines 786-O and Caki-1 with miR-182-5p mimics and examined the effects on cellular proliferation. The ectopic expression of miR-182-5p was confirmed by qRT-PCR (Additional file 2: Figure S2). CCK-8 and colony formation assays revealed that the overexpression of miR-182-5p significantly decreased the growth rate of both RCC cell lines, compared to NC-transfected cells (Figure 2A and B). To further confirm the above findings, the growth rates of Caki-1 cells with or without miR-182-5p over-expression were examined after s.c. implantation into BALB/c mice. The over-expression of miR-182-5p resulted in a dramatic retardation of tumor growth *in vivo* (Figure 2C and D). IHC staining confirmed that the tumors derived from the miR-182-overexpressing cells displayed much lower Ki-67 indices than the tumors from the control group (Figure 2E). Taken together, these results showed that miR-182-5p negatively modulate RCC cells growth.

### Upregulation of miR-182-5p in RCC cells triggers G1-phase arrest and regulates cell cycle factors through AKT/FOXO3a signaling

**Figure 3:**
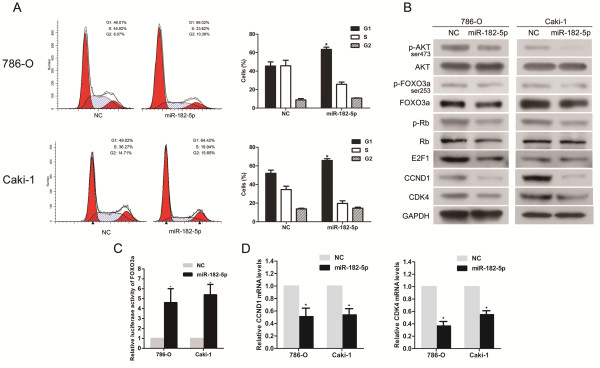
**Overexpression of miR-182-5p inhibits the G1/S transition and cell cycle progression in RCC cells. (A)** Flow cytometric analysis of cell cycle distribution. Over-expression of miR-182-5p induced a significant accumulation of cells in G1-phase and blocks G1-S entry. **(B)** Western blotting analysis of indicated proteins. **(C)** FOXO3a activity was strongly activated by upregulation of miR-182-5p. **(D)** Real-time PCR analysis of the expression of CCND1 and CDK4 mRNA; GAPDH was used as a loading control. Error bars represent the S.D. from three independent experiments. *P < 0.05.

The underlying mechanism for miR-182-5p-suppressed tumor growth was further explored with FACS. We observed a significant increase in the percentage of cells in the G1/G0 phase and a decrease in the percentage of cells in the S phase in miR-182-5p-overexpressing cells (Figure 3A). Consistent with the cell cycle arrest phenomenon, the G1/S transition regulators CCND1 and CDK4 were significantly decreased at the protein and mRNA levels in miR-182-5p-overexpressing cells (Figure 3B, 3D and Additional file 3: Figure S3). p-Rb and E2F1, the major downstream effector proteins of cell cycle signaling, also showed obvious changes in expression (Figure 3B and Additional file 3: Figure S3). It has been well documented that the expression of CCND1 can be transcriptionally regulated by FOXO3a [33] and, in turn, the transcriptional activity of FOXO3a is modulated by AKT phosphorylation [34,35]. To further confirm that FOXO3a is a downstream target of AKT phosphorylation in RCC cells, we found that a small molecule inhibitor of AKT (LY294002) could significantly activate FOXO3a (Additional file 4: Figure S4). Thus, we hypothesized that the upregulation of miR-182-5p might inhibit AKT/FOXO3a signaling. As shown in Figure 3B and Additional file 3: Figure S3, the phosphorylation levels of both FOXO3a and AKT decreased in miR-182-5p-overexpressing RCC cells. In addition, FOXO3a activity was strongly activated by the upregulation of miR-182-5p, as demonstrated by a FOXO3a luciferase reporter vector (Figure 3C).

### miR-182-5p downregulates FLOT1 expression by directly targeting its 3′UTR

**Figure 4:**
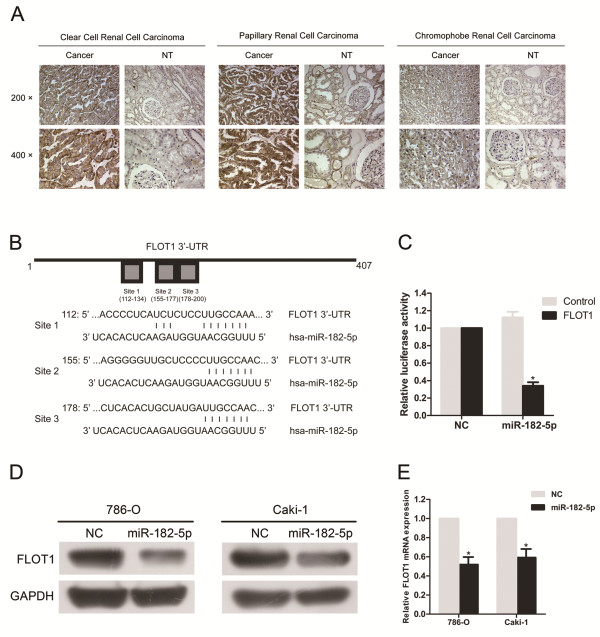
**FLOT1 is a direct target of miR-182-5p. (A)** Representative IHC analyses of FLOT1 expression in normal kidney tissue and RCC specimens of three types of RCC. **(B)** Predicted miR-182-5p target sequences in the 3′-UTR of FLOT1. **(C)** miR-182-5p significantly suppressed the luciferase activity of vector that carried 3′-UTR of FLOT1 but not control vector. **(D)** Western blot analysis confirmed that miR-182-5p inhibited the endogenous expression of FLOT1. **(E)** FLOT1 mRNA levels decreased when miR-182-5p was up-regulated. Error bars represent the S.D. from three independent experiments. *P < 0.05.

A miRNA usually performs its function by reducing the expression of target genes. Thus our next aim was to investigate the targets of miR-182-5p that contributed to its anti- proliferation function. FLOT1, a putative target of miR-182-5p identified by TargetScan, was of particular interest because it had three high scoring predicted binding sites and was previously considered as a positive cell cycle regulator in breast cancer [36]. In our current study, we revealed that FLOT1 was commonly over-expressed in all three types of renal cell cancer tissues (Figure 4A and Additional file 5: Figure S5). With qRT-PCR and western blot, we verified that FLOT1 was significantly decreased in both mRNA and protein level after the over-expression of miR-182-5p (Figure 4D and E).

We then carried out luciferase reporter assays to verify a direct interaction between miR-182-5p and the 3′UTR of FLOT1. The 3′-UTR of FLOT1 mRNA has 3 putative miR-182-5p binding sites (Figure 4B). We cloned the 3′-UTR into down-stream of firefly luciferase of pmirGLO Dual-Luciferase miRNA Target Expression Vector. Cotransfected of either miR-182-5p or NC and luciferase reporter constructs comprising 3′-UTR was conducted. HEK 293 T cells transiently transfected with the 3′- UTR-reporter and miR-182-5p showed significantly decreased relative luciferase activity when compared with NC. However, the luciferase activity of the control vector was unaffected by the simultaneous transfection of miR-182-5p (Figure 4C).

### Repression of FLOT1 plays essential roles in miR-182-5p-supressed proliferation of RCC cells

**Figure 5:**
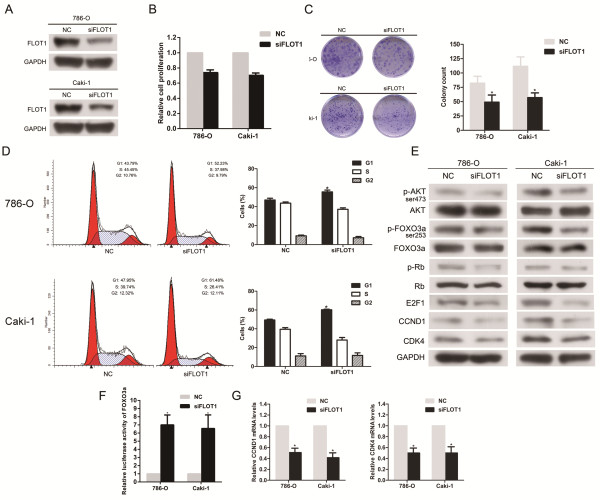
**Downregulation of FLOT1 phencopied the effect of miR-182-5p. (A)** Western blot analysis showed reduction of FLOT1 protein after siFLOT1 treatment. **(B)** CCK-8 assay. The relative cell viability of the siFLOT1 transfected group was significantly lower than that of NC transfected. **(C)** Colony formation assay (Representative wells were presented). The colony formation rate was significantly lower for siFLOT1 treated group compared with NC treated group. **(D)** Flow cytometric analysis of cell cycle distribution. Down expression of FLOT1 induced a significant accumulation of cells in G1-phase and blocks G1-S entry. **(E)** Western blotting analysis of indicated proteins. **(F)** FOXO3a activity was strongly activated by downregulation of FLOT1. **(G)** Real-time PCR analysis of the expression of CCND1 and CDK4 mRNA; GAPDH was used as a loading control. Error bars represent the S.D. from three independent experiments. *P < 0.05.

**Figure 6:**
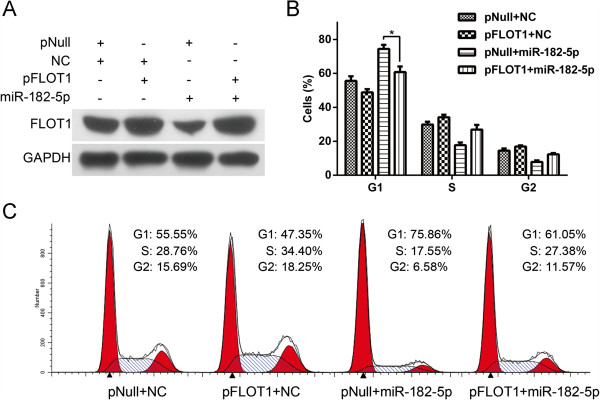
**Forced expression of FLOT1 partly rescued miR-182-5p-dependent G1 phase arrest. (A)** Caki-1 cells were co-transfected with either miR-182-5p mimics or NC oligos with pIRES-EGFP-FLOT1 or empty pIRES-EGPF vector. The expression of FLOT1 or GAPDH was detected by Western blot analysis. **(B)** and **(C)** Forced expression of FLOT1 partly abrogated cell cycle arrest effect of miR-182-5p in Caki-1 cells. Error bars represent the S.D. from three independent experiments. *P < 0.05.

To determine whether the downregulation of FLOT1 was involved in miR-182-5p-mediated suppression of proliferation, we first analyzed the functions of FLOT1 in RCC cells, which had not been previously reported. As shown in Figure 5A and Additional file 6: Figure S6, the transfection of small interfering RNA against FLOT1 into 786-O and Caki-1 cells led to dramatically decreased FLOT1 expression in protein and mRNA levels. Moreover, silencing FLOT1 significantly suppressed the proliferation and tumorigenicity of RCC cells *in vitro* and induced G1 arrest (Figure 5B, C and D), which phenocopied the effects of miR-182-5p on RCC cells. In addition, silencing FLOT1 also inhibited AKT/FOXO3a signaling. As shown in Figure 5E, the phosphorylation levels of both FOXO3a and AKT decreased in FLOT1-knockdown RCC cells, and FOXO3a activity was strongly induced (Figure 5F and Additional file 7: Figure S7). Accordingly, CCND1, CDK4, p-Rb and E2F1 expression levels were significantly decreased (Figure 5E and Additional file 7: Figure S7). In parallel, co-transfection of pFLOT1 was applied to abrogate the FLOT1 expression inhibition by miR-182-5p (Figure 6A). Forced FLOT1 expression partially, but significantly, attenuated the G1-phase arrest induced by miR-182-5p (Figure 6B and C) and promoted cell viability (Additional file 8: Figure S8).

## Discussion

In recent years, emerging studies have clearly confirmed the important roles of miRNAs in the tumorigenesis of RCC and other malignancies. Although many studies have characterized the miRNA signatures of RCC, the roles of miRNA dysregulation in RCC proliferation and tumorigenicity remain elusive. In the present study, the miR-182-5p expression level was significantly lower in RCC tissues compared with the corresponding non-tumor tissues. Furthermore, gain-of-function analyses of miR-182-5p *in vitro and in vivo* suggest that miR-182-5p was able to suppress the proliferation and tumorigenicity of RCC cells, and may serve as a tumor-suppressor gene.

The human miR-182-5p, located at chromosome 7q32 region, is transcribed from the cluster of the miR-183 family and has been extensively researched in human cancers. On one hand, miR-182-5p was reported to function as an oncogene in most common types of human cancers, but on the other hand, miR-182-5p exhibited tumor-suppressive activity in human gastric adenocarcinoma, lung adenocarcinomas, and posterior uveal melanoma [26-28]. MicroRNAs have been shown to frequently regulate numerous target genes with possible counteracting functions. Thus, the cell context-dependent balance among the network of directly regulated genes of miR-182-5p may determine the biological function in a specific cancer. In this study, we observed that the expression of miR-182-5p was decreased in most of our RCC tissues and that miR-182-5p may serve as a tumor-suppressor gene. Although down expression of miR-182-5p has been reported in several types of human cancers [26-28], little is known about the mechanisms of its downregulation. Thus, the first important finding of our present study is that epigenetic modulation may be involved in the regulation of miR-182 expression.

We have also demonstrated that the molecular mechanism by which miR-182-5p inhibited RCC cell proliferation and tumorigenicity could be attributed to, at least in part, triggering G1-phase arrest via suppression of the AKT/FOXO3a signaling pathway. Previous research has indicated that the PI3K/AKT pathway plays an important role in cell cycle progression through the G1 phase [37].The activation of PI3K/AKT reduces the levels of p21Cip1 and p27Kip1, and increases expression of CCND1 [38,39], thereby promoting cell proliferation. FOXO3a is a major PI3K/AKT effector in human cancers [34,35,40] and transcriptionally regulates the above mentioned cell cycle regulators [33]. In the current study, we found that the upregulation of miR-182-5p decreased AKT phosphorylation and significantly increased the transactivation activity of FOXO3a, suggesting that this pathway might represent a new mechanism underlying the development of RCC.

A miRNA usually performs its specific function by decreasing the expression of target genes. Thus, we further explored the possible mechanism by which miR-182-5p could inhibit the AKT/FOXO3a signaling pathway. As a result, FLOT1, a marker of lipid rafts, was identified as a novel target of miR-182-5p. FLOT1 encodes a caveolae-associated, integral membrane protein that belongs to lipid raft family members and functions as a signaling molecule that tethers membrane receptors linked to signal transduction pathways in various types of cells [41,42]. It has been reported that the silencing of FLOT1 inhibited the proliferation and tumorigenesis of breast cancer, oral squamous cell carcinoma and esophageal squamous cell carcinoma cells both in *vitro and in vivo*[36,43,44]. Moreover, in breast cancer, esophageal squamous cell carcinoma, hepatocellular carcinoma, and lung adenocarcinoma, overexpression of FLOT1 could be used as a valuable maker for the prediction of a poor prognosis for patients [36,44-46]. All of these findings suggested an oncogenic role for FLOT1 in human cancers.

Regarding RCC, Raimondo’s membrane proteomic study suggested that FLOT1 was up-regulated in human RCC tissues compared to para-cancer kidney tissues [47]. However, until now, the function of FLOT1 in RCC has not been reported. In this study, we demonstrated that FLOT1 was overexpressed in RCC versus adjacent non-tumor tissues and that silencing FLOT1 significantly inhibited the proliferation and tumorigenesis of RCC cells through regulation of the AKT/FOXO3a pathway. Additionally, FLOT1 was directly regulated by miR-182-5p. Ectopic overexpression of FLOT1 (without the 3′-UTR) significantly abrogated the miR-182-induced G1 arrest of RCC cells and promoted cell viability *in vitro*. Taken together, these results suggest that miR-182-5p inhibits the proliferation of RCC cells via, at least in part, directly targeting the 3′-UTRs of FLOT1. Thus, our current study reveals what we believe to be a novel upstream regulatory mechanism of FLOT1 in cancer cells

## Conclusions

Our study suggests that miR-182-5p is a potential tumor suppressor in RCC. miR-182-5p, by targeting FLOT1, could suppress proliferation and tumorigenesis of RCC cells. The restoration of miR-182-5p could be a vigorous therapeutic strategy for RCC treatment.

## Materials and methods

### Cell lines and cell culture

The human RCC cell lines 786-O and Caki-1 were purchased from the Shanghai Institute of Cell Biology, Shanghai, China and were cultured in RPMI 1640 medium supplemented with 10% heat-inactivated fetal bovine serum under a humidified atmosphere of 5% CO_2_ at 37°C.

### Tissue samples

Twenty-five paired renal cancer tissues and adjacent non-tumor tissues were obtained from patients undergoing radical nephrectomy. The samples were collected between January 2013 and October 2013 at the First Affiliated Hospital of Medical College, Zhejiang University (Hangzhou, P.R. China) after informed consent and the approval of the Ethics Committee of Zhejiang University. Clinicopathological characteristics of the patients are presented in Supporting Additional file 9: Table S1. Additionally, a commercial tissue microarray bearing 31 pairs of renal cell cancer and corresponding non-tumor tissues (10 clear cell renal cell carcinoma, 11 papillary renal cell carcinoma and 10 chromophobe renal cell carcinoma, respectively) were purchased from Shanghai Outdo Biotech for immunohistochemical analysis of FLOT1.

### RNA isolation and qRT-PCR

Total RNA was extracted using RNAiso plus (Takara, Japan) according to the manufacturer’s instructions. Reverse transcription reactions were performed using PrimeScript RT reagent Kit (Takara, Japan) for mRNA detection. The resulting cDNA was quantified with the ABI 7500 FAST real-time PCR System (Applied Biosystems, Carlsbad, USA) using SYBR Green (Takara, Dalian, China). Levels of relative expression were calculated and quantified with the 2^-∆∆Ct^ method after normalization with reference to expression of GAPDH.

To detect the level of miR-182-5p, the complementary DNA was synthesized using One Step PrimeScript miRNA cDNA Synthesis Kit (TaKaRa, Japan), and real-time PCR analysis was performed with the ABI 7500 FAST real-time PCR System. The expression of human U6 small nuclear RNA was used for normalization. All primers used were listed in Table 1.

**Table 1:** The oligonucleotides used in this study

**Name**^**a**^	**Sequence(5′->3′)**^**b**^
miR-182-5p mimics (sense)	UUUGGCAAUGGUAGAACUCACACU
NC (sense)	ACTACTGAGTGACAGTAGA
miR-182-5p F	TTTGGCAATGGTAGAACTCACACT
U6 F	TGCGGGTGCTCGCTTCGGCAGC
FLOT1 F	CCCATCTCAGTCACTGGCATT
FLOT1 R	CCGCCAACATCTCCTTGTTC
CCND1 F	GCTGCGAAGTGGAAACCATC
CCND1 R	CCTCCTTCTGCACACATTTGAA
CDK4 F	ATGGCTACCTCTCGATATGAGC
CDK4 R	CATTGGGGACTCTCACACTCT
GAPDH F	AAGGTGAAGGTCGGAGTCA
GAPDH R	GGAAGATGGTGATGGGATTT
FLOT1-utr F	TCGA**GAGCTC**GCTGAAGTTGCCTGAATGAT
FLOT1-utr R	TCGA**GTCGAC**AGCCCATCCCTCAGTCTT

^a^F, forward primer; R, reverse primer.

^b^Restriction sites are in bold.

### Immunohistochemistry (IHC) staining

IHC staining was conducted as described previously [48]. Briefly, tissue sections were dewaxed and rehydrated before performing antigen retrieval. The slides were incubated with anti-ki-67 or anti-FLOT1 (Epitomics, Burlingame, USA) overnight at 4°C, and incubated with an HRP-conjugated secondary antibody for 1 h at room temperature. DAB was used for color development, and dark brown staining was considered positive. The strength of positivity was semi-quantified by taking into account the staining intensity and the percentage of positive cells.

### Transient transfection of miRNA mimics and small interfering RNAs

The miR-182-5p mimic, small interfering RNAs (siRNAs) of FLOT1 and the negative control were purchased from GenePharma (Shanghai, China). For convenience, these are termed miR-182-5p, siFLOT1 and NC, respectively. miRNA and siRNA transfection was carried out using Lipofectamine 2000 (Invitrogen, Carlsbad, CA, USA) in accordance with the manufacturer’s procedure.

### Cell proliferation assay

Approximately 4 × 10^3^ 786-O cells or Caki-1 cells were plated in each well of a 96-well plate. After an overnight incubation, the cells were transfected with the RNAs (miR-182-5p, siFLOT1 or NC) for 2 days. Then, the medium was removed, and cell counting solution (WST-8, Dojindo Laboratories, Tokyo, Japan) was added to each well and incubated for an additional 1 h. The absorbance of the solution was measured spectrophotometrically at 450 nm with MRX II absorbance reader (Dynex Technologies, Chantilly, VA, USA).

### *In vitro* colony formation assay

Cells were trypsinized to single cell suspensions 24 h after transfection with 2′-O-Methyl modified duplexes (50 nM). Then, the cells were seeded into fresh six-well plates at 600 cells/well. After 2 weeks, the colonies were fixed with absolute methanol and then stained with crystal violet. The colonies with a diameter over 2 mm were counted.

### *In vivo* tumorigenicity assays

Animal studies were carried out according to institutional guidelines. Male BALB/c-nude mice (4 weeks old) were purchased from the Shanghai Experimental Animal Center, Chinese Academy of Sciences, Shanghai, China. Caki-1 cells (1 × 10^6^ in 100 μl PBS) were injected subcutaneously into the right flank of each mouse. When palpable tumors arose, the mice were injected intratumorally with 30 μg of Lipofectamine 2000-encapsulated miR-182-5p or NC every 3 days for 3 weeks. Tumor size was monitored and evaluated every 3 days. Tumor growth was monitored by caliper measurements of the two perpendicular diameters every 3 days, and the volume of the tumor was calculated with the formula V = (width^2^ × length × 0.52).

### Cell cycle analysis by flow cytometry

Cells were collected 48 h after RNA treatment and fixed in 75% ethanol overnight at −20°C. Then, the cells were washed twice with PBS and followed by RNase A and propidium iodide (50 μg/ml) treatment for 30 min. Finally, cell cycle analyses were performed with the BD LSRII Flow Cytometer System with FACSDiva Software (BD Bioscience, Franklin Lakes, USA). The raw data were analyzed by ModFit LT 3.2 software (Verity Software House, Topsham, USA).

### 5-Aza-2′-deoxycytidine treatment of the 786-O and Caki-1 cell line

786-O and Caki-1 cells were treated with 5 μM 5-Aza-2′-deoxycytidine (Sigma A3656) for 4 days. RNA was extracted and analyzed for the expression of miR-182-5p.

### DNA methylation analysis

Genomic DNA from 786-O and Caki-1 cell line and two pairs of RCC and corresponding non-tumor tissues was bisulfite modified and the CpG islands amplified by PCR using the primers (forward) 5′- GTTAYGATGAGGTTATTAGGATAGAT -3′; (reverse) 5′- ATATCCTCCAAACTAAACCATTC -3′. The PCR products were separated by 3% agarose gel electrophoresis, extracted and then cloned into the pUC18 T-vector (Sangon, China). After bacterial amplification of the cloned PCR fragments by standard procedures, 8 clones were subjected to DNA sequencing (Sangon, China).

### Western blot analysis

Western blot analysis was carried out as previously described [49] with the following appropriate primary immunoblotting antibodies: anti-GAPDH, anti-FLOT1, anti-E2F1, anti-CDK4, anti-FOXO3a, anti-p-FOXO3a (phospho S253), anti-RB, anti-p-RB, anti-p-AKT (phospho S473) (Epitomics, Burlingame, CA), anti-AKT, anti-CCND1 (Cell Signaling Technology, Beverly, MA).

### Vector construction and dual-luciferase reporter assay

The 3′-UTR of FLOT1 was cloned downstream of the luciferase reporter in the pmirGLO Dual-Luciferase miRNA Target Expression Vector (Promega, Madison, USA) between the SacI and SalI sites and verified by sequencing. HEK 293 T cells were plated in 24-well plates and transfected with 50 nM miR-182-5p or NC and 100 ng of the luciferase vector (pmirGLO). Cells were harvested 48 h post-transfection. The relative luciferase activity was measured by the Dual-Glo luciferase assay kit (Promega).

### FLOT1 rescue experiments

The FLOT1 plasmid was constructed by inserting the human FLOT1 complementary DNA lacking the 3′-UTR into the pIRES2-EGFP (Clontech, USA, the over-expression clone of FLOT1 would be termed as pFLOT1). miR-182-5p or NC was cotransfected with pFLOT1 or the empty vector (pNull). The cells were harvested 48 h after transfection and analyzed with subsequent Western blotting and cell cycle analysis.

### Statistical analysis

The data were expressed as the mean ± SD. All analyses were performed using GraphPad Prism version 5 for Windows and a two-tailed value of *P* < 0.05 was considered statistically significant with Student’s t-test.

## Abbreviations

RCC: Renal cell carcinoma; UTR: Untranslated region; CCK-8: Cell Counting Kit-8; IHC: Immunohistochemistry; miRNA: MicroRNA; qRT-PCR: Quantitative-RT-PCR; ncRNA: Non-coding RNAs; BSP: Bisulfite sequencing PCR; TSS: Transcription start site.

## Competing interest

The authors declare that they have no competing interest.

## Authors’ contributions

XX, JW, SL, ZH, XX, YZ, and XW performed experiments; LX, XZ, BL, JL, ZL, YL, HC, and YM designed research, analyzed data and edited the manuscript for intellectual content. All authors read and approved the final manuscript.

## Supplementary Material

Additional file 1: Figure S1 The treatment of 786-O and Caki-1 cells with 5-Aza apparently reduced the methylation level of this CpG island. Error bars represent the S.D. from eight randomly chosen colonies. Click here for file

Additional file 2: Figure S2 The ectopic expression of miR-182-5p was confirmed by qRT-PCR. Error bars represent the S.D. from three independent experiments. *P < 0.05. Click here for file

Additional file 3: Figure S3 Expression levels were quantitated using ImageJ software (Wayne Rashband); GAPDH was used as a loading control. Error bars represent the S.D. from three independent experiments. *P < 0.05. Click here for file

Additional file 4: Figure S4 LY294002 significantly activated FOXO3a. (A) Western blotting analysis of indicated proteins. (B) Relative FOXO3a reporter activity was strongly activated. Error bars represent the S.D. from three independent experiments. *P < 0.05. Click here for file

Additional file 5: Figure S5 Positive strength of FLOT1 was significantly higher in RCC tissues compared with paired non-tumor tissues. Error bars represent the S.D. from different patients. ***P < 0.001. Click here for file

Additional file 6: Figure S6 Expression of FLOT1 after siFLOT1 treatment was detected by qRT-PCR. Error bars represent the S.D. from three independent experiments. *P < 0.05. Click here for file

Additional file 7: Figure S7 Expression levels were quantitated using ImageJ software (Wayne Rashband); GAPDH was used as a loading control. Error bars represent the S.D. from three independent experiments. *P < 0.05. Click here for file

Additional file 8: Figure S8 Forced expression of FLOT1 increase cell viability. Error bars represent the S.D. from three independent experiments. *P < 0.05. Click here for file

Additional file 9: Table S1 Patients and tumor characteristics (n = 25). Click here for file
