# Supplementary figures and images for: Downregulation of microRNA-182-5p contributes to renal cell carcinoma proliferation via activating the AKT/FOXO3a signaling pathway
Source: Mol Cancer. 2014 May 17;13:109. doi: 10.1186/1476-4598-13-109 (PMC4040501; doi:10.1186/1476-4598-13-109)

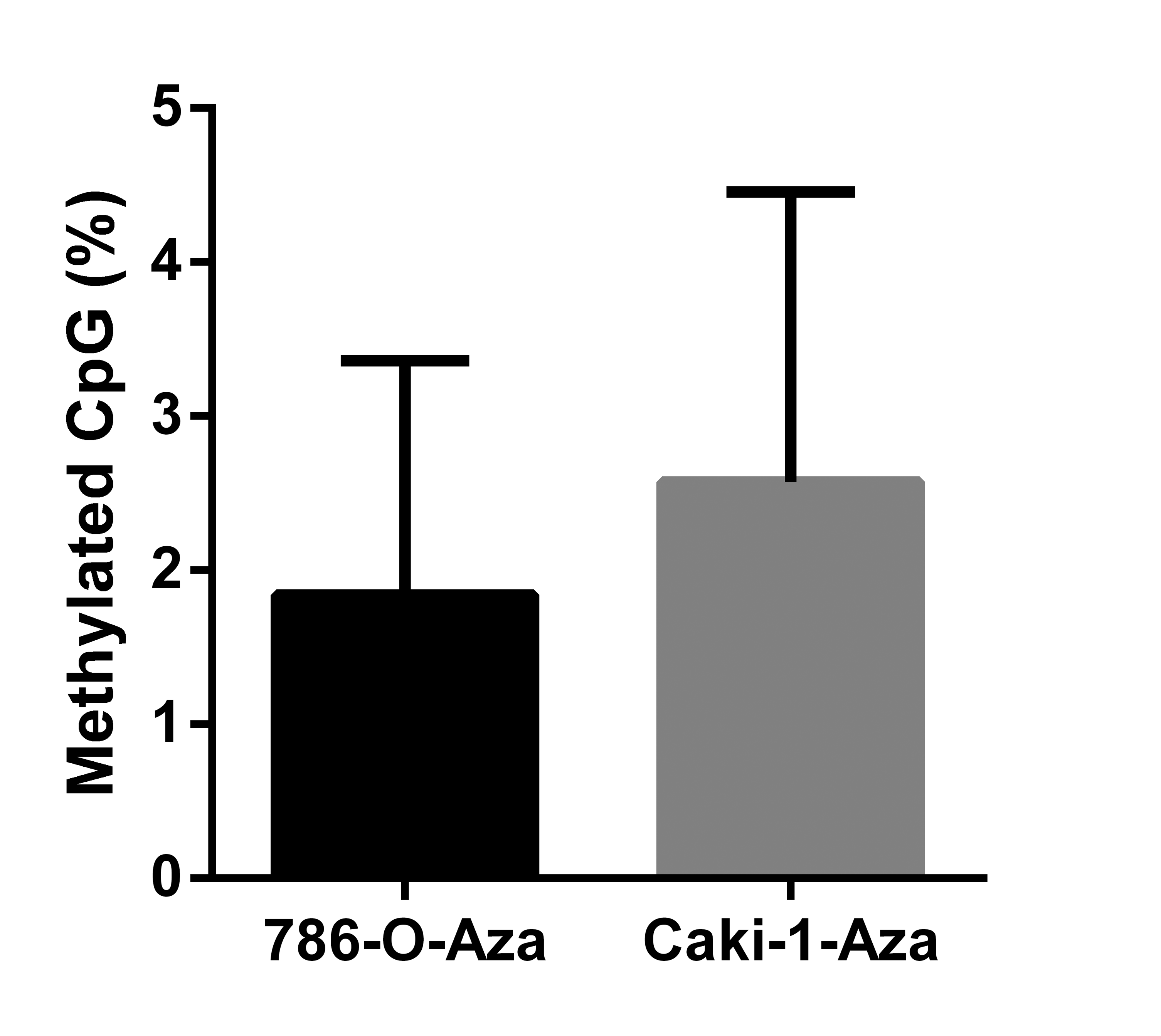

Supplement: Additional file 1: Figure S1 — The treatment of 786-O and Caki-1 cells with 5-Aza apparently reduced the methylation level of this CpG island. Error bars represent the S.D. from eight randomly chosen colonies. [file 1476-4598-13-109-S1.tiff]

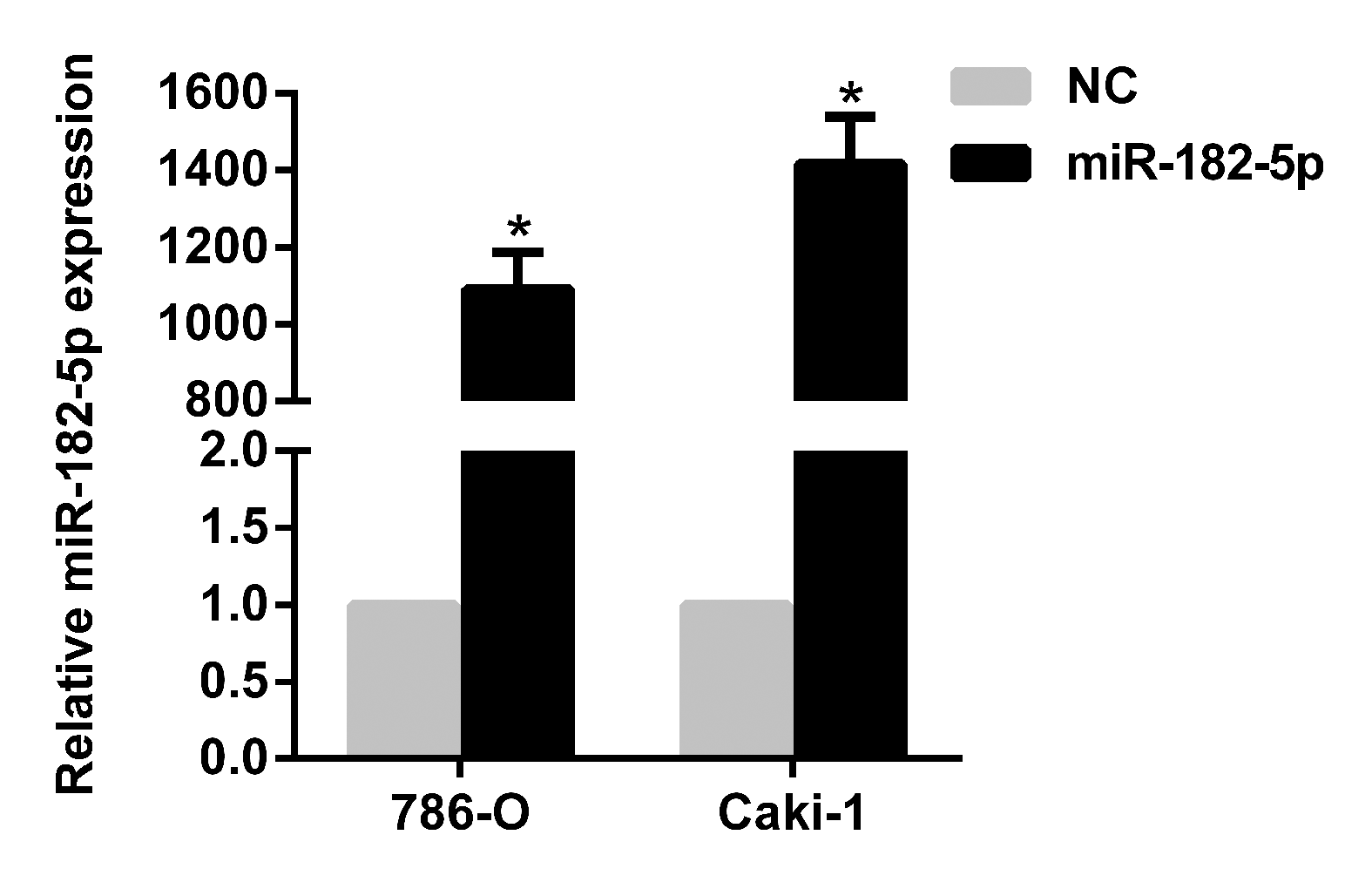

Supplement: Additional file 2: Figure S2 — The ectopic expression of miR-182-5p was confirmed by qRT-PCR. Error bars represent the S.D. from three independent experiments. *P < 0.05. [file 1476-4598-13-109-S2.tiff]

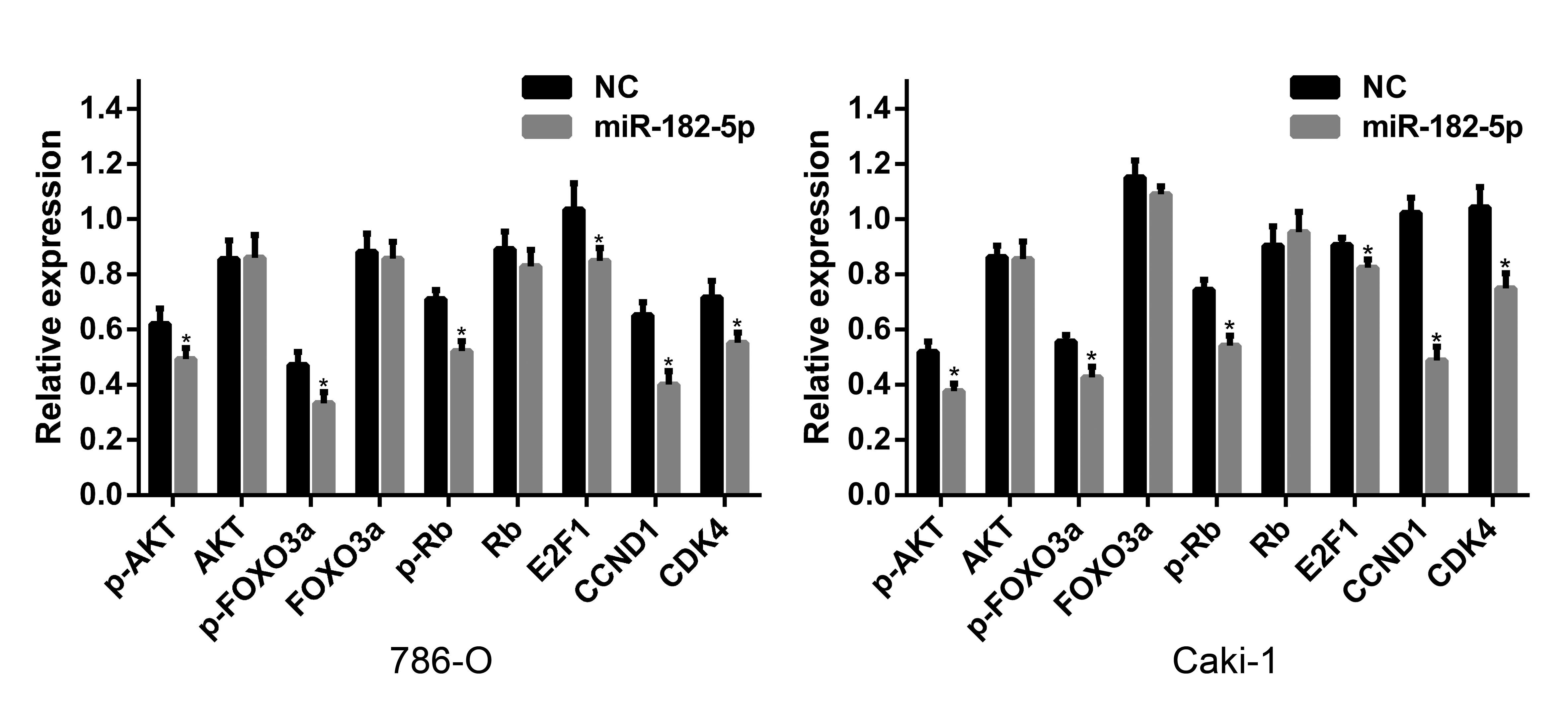

Supplement: Additional file 3: Figure S3 — Expression levels were quantitated using ImageJ software (Wayne Rashband); GAPDH was used as a loading control. Error bars represent the S.D. from three independent experiments. *P < 0.05. [file 1476-4598-13-109-S3.tiff]

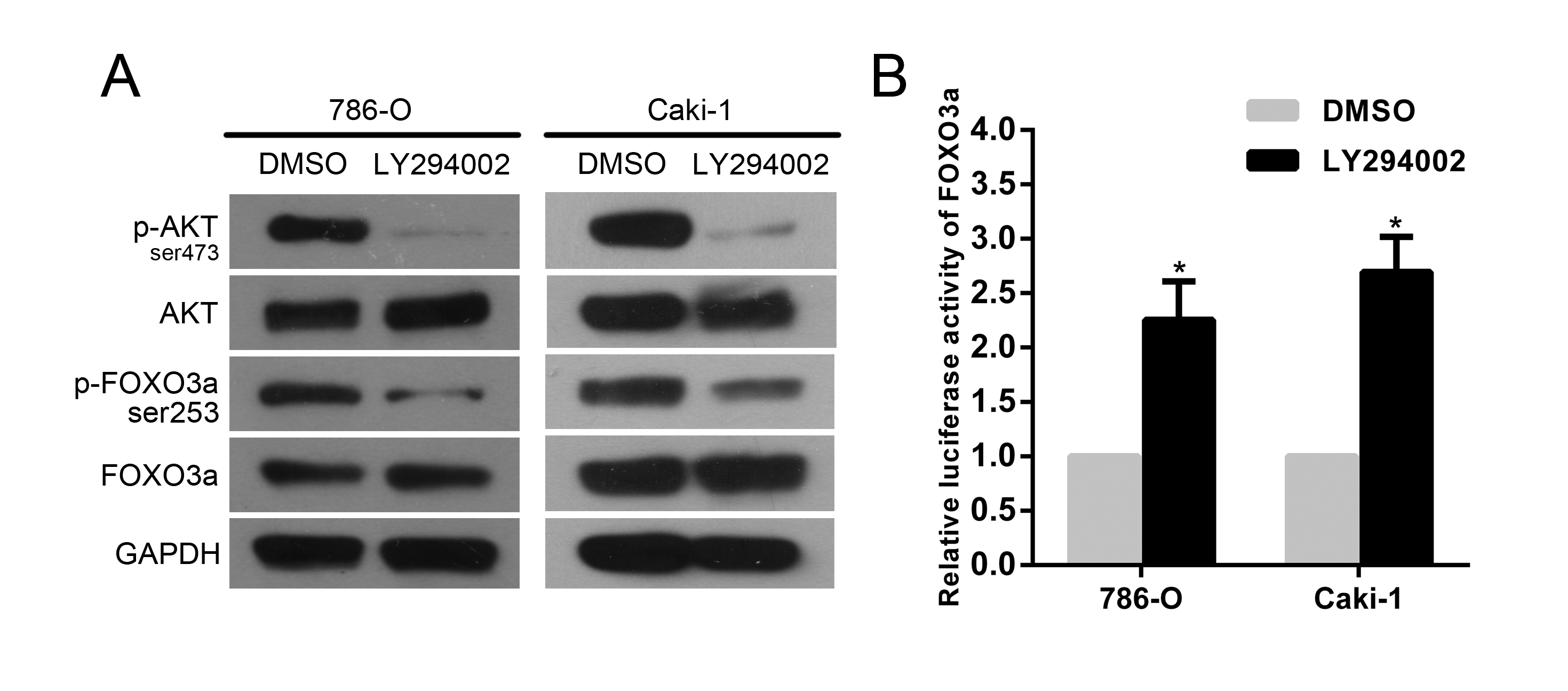

Supplement: Additional file 4: Figure S4 — LY294002 significantly activated FOXO3a. (A) Western blotting analysis of indicated proteins. (B) Relative FOXO3a reporter activity was strongly activated. Error bars represent the S.D. from three independent experiments. *P < 0.05. [file 1476-4598-13-109-S4.tiff]

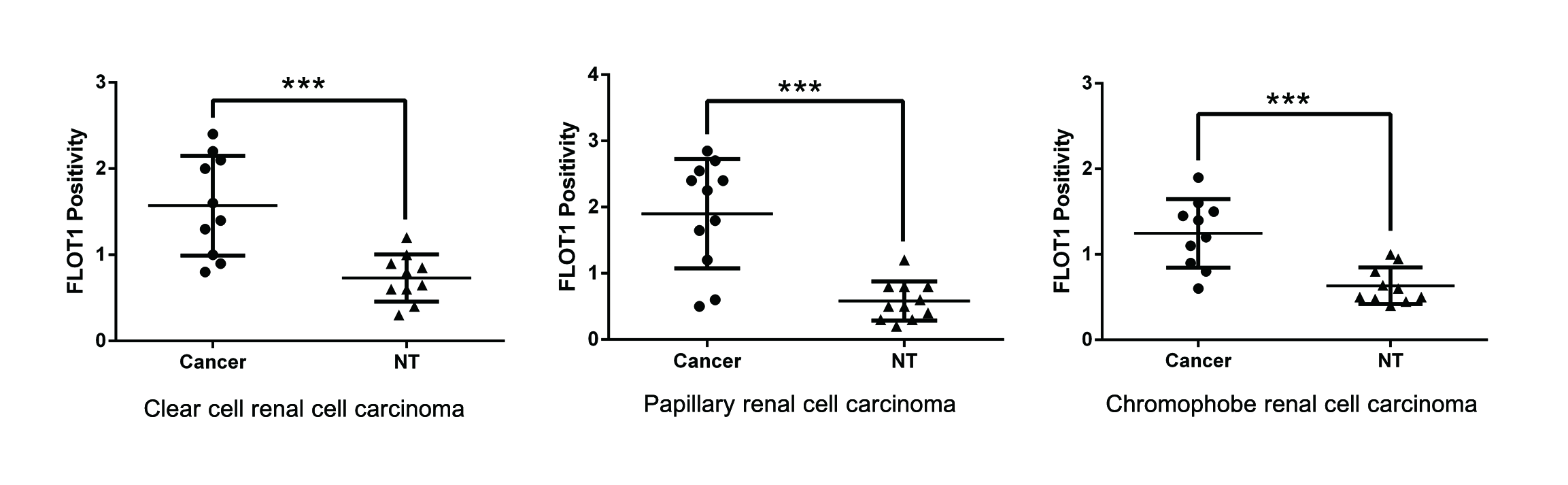

Supplement: Additional file 5: Figure S5 — Positive strength of FLOT1 was significantly higher in RCC tissues compared with paired non-tumor tissues. Error bars represent the S.D. from different patients. ***P < 0.001. [file 1476-4598-13-109-S5.tiff]

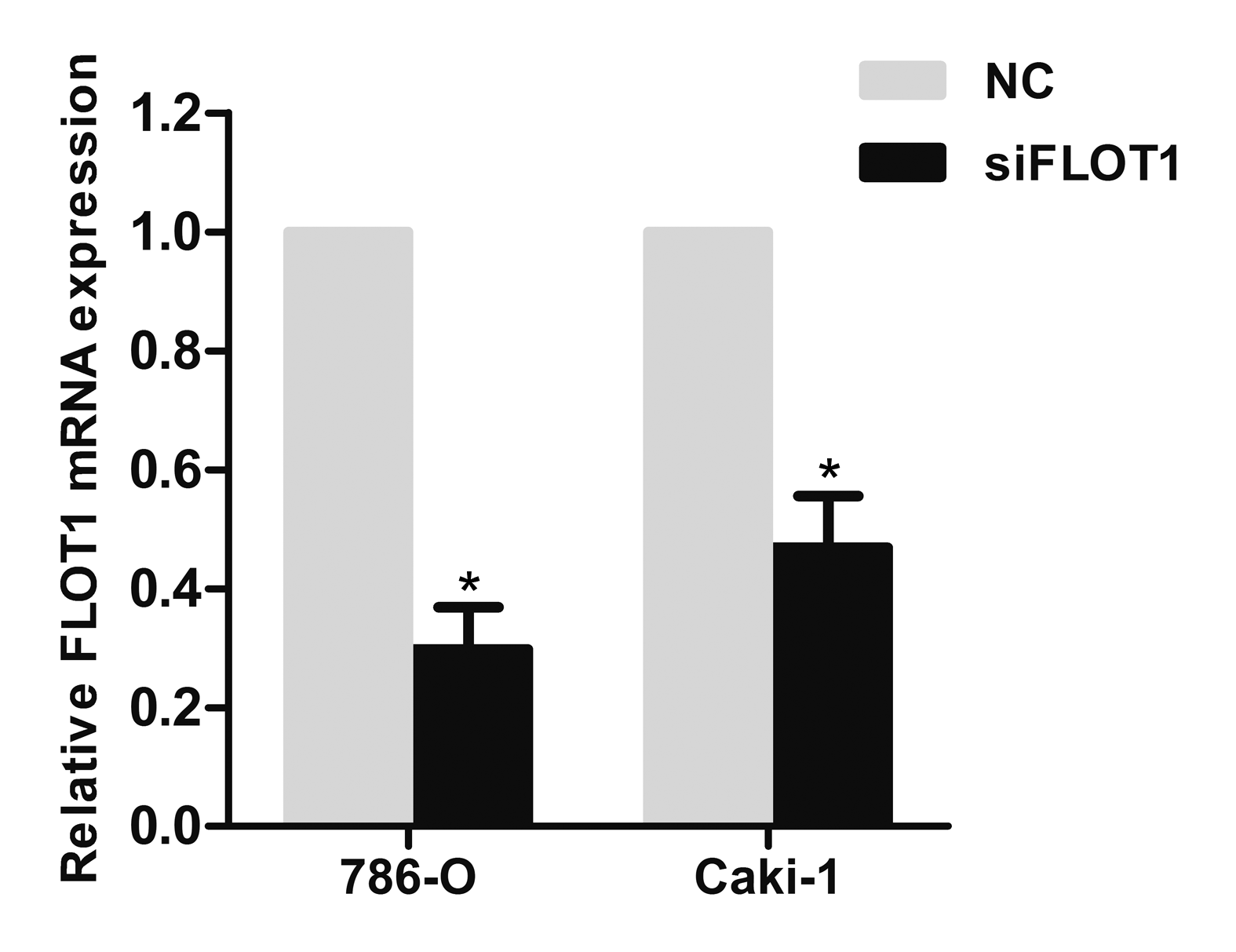

Supplement: Additional file 6: Figure S6 — Expression of FLOT1 after siFLOT1 treatment was detected by qRT-PCR. Error bars represent the S.D. from three independent experiments. *P < 0.05. [file 1476-4598-13-109-S6.tiff]

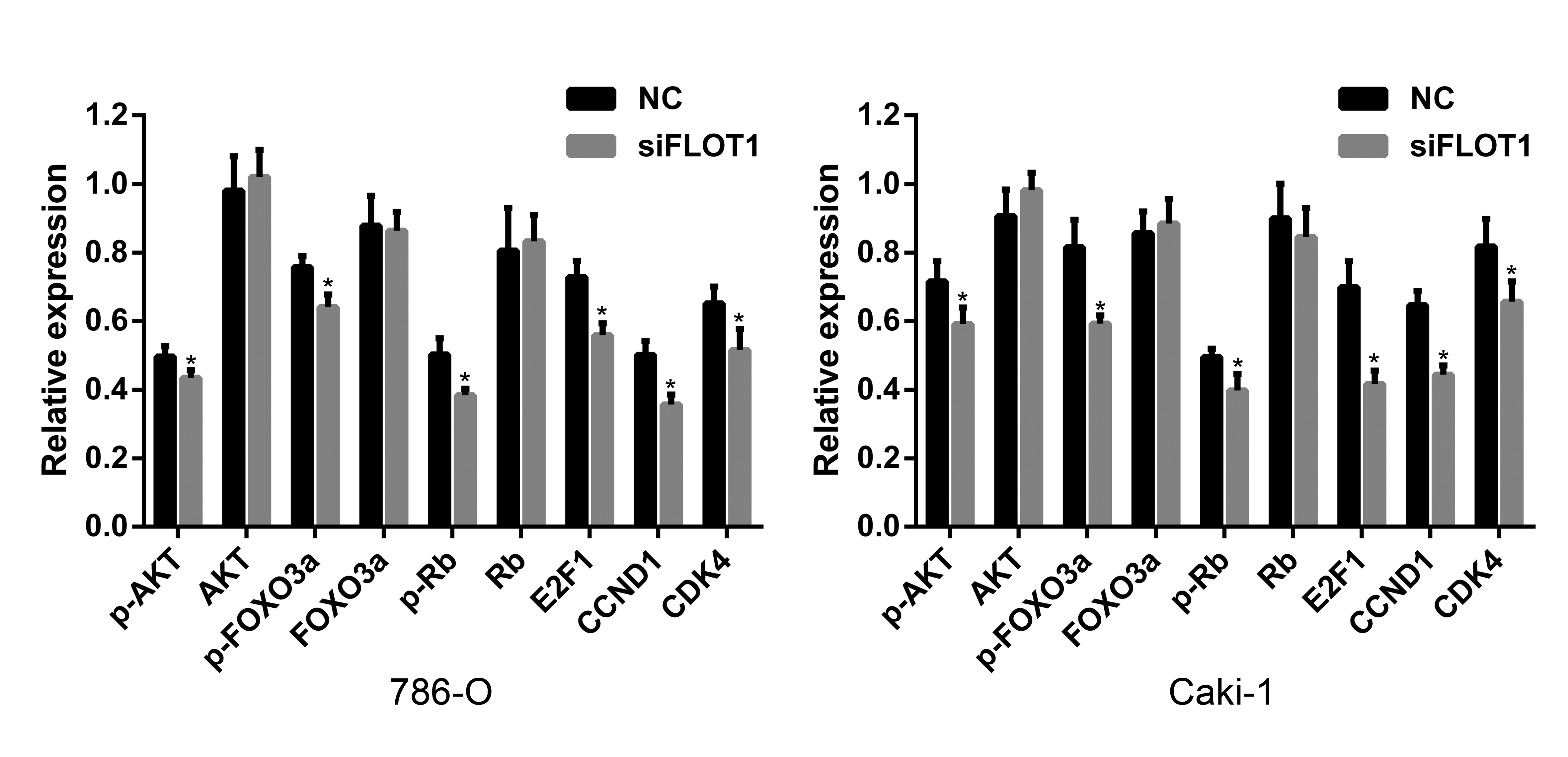

Supplement: Additional file 7: Figure S7 — Expression levels were quantitated using ImageJ software (Wayne Rashband); GAPDH was used as a loading control. Error bars represent the S.D. from three independent experiments. *P < 0.05. [file 1476-4598-13-109-S7.tiff]

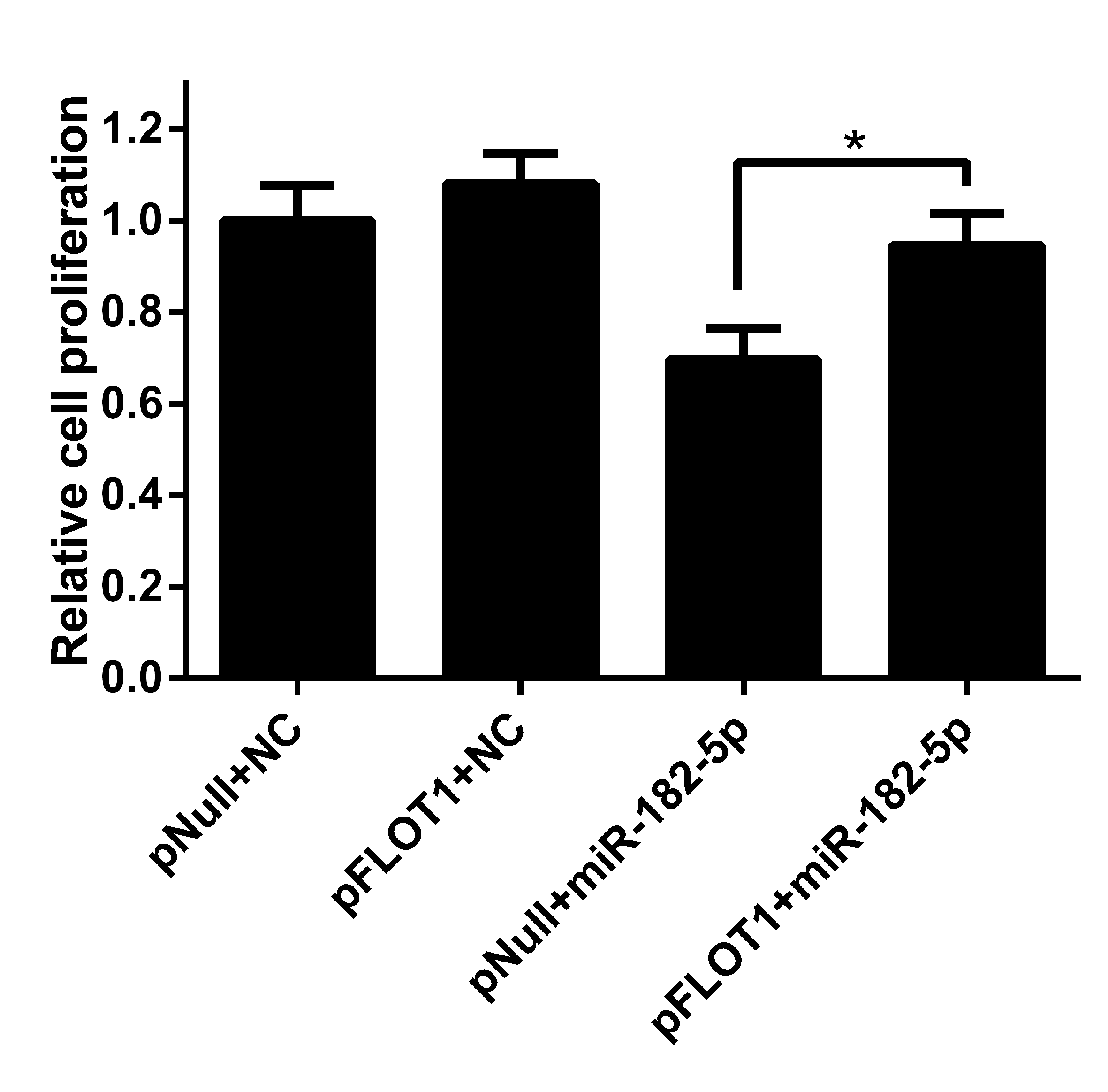

Supplement: Additional file 8: Figure S8 — Forced expression of FLOT1 increase cell viability. Error bars represent the S.D. from three independent experiments. *P < 0.05. [file 1476-4598-13-109-S8.tiff]
